# Coding linguistic elements in clinical interactions: a step-by-step guide for analyzing communication form

**DOI:** 10.1186/s12874-022-01647-0

**Published:** 2022-07-11

**Authors:** Inge Stortenbeker, Lisa Salm, Tim olde Hartman, Wyke Stommel, Enny Das, Sandra van Dulmen

**Affiliations:** 1 Centre for Language Studies, Radboud University, Nijmegen, the Netherlands; 2 Radboud university medical center, Radboud Institute for Health Sciences, Department of Primary and Community Care, Nijmegen, the Netherlands; 3 NIVEL (Netherlands institute for health services research), Utrecht, the Netherlands; 4 Faculty of Caring Science, University of Borås, Borås, Sweden

**Keywords:** Provider-patient interactions, Language use, Codebook development, Quantifying communication

## Abstract

**Background:**

The quality of communication between healthcare professionals (HCPs) and patients affects health outcomes. Different coding systems have been developed to unravel the interaction. Most schemes consist of predefined categories that quantify the content of communication (the *what*). Though the form (the *how*) of the interaction is equally important, protocols that systematically code variations in form are lacking. Patterns of form and how they may differ between groups therefore remain unnoticed. To fill this gap, we present CLECI, Coding Linguistic Elements in Clinical Interactions, a protocol for the development of a quantitative codebook analyzing communication form in medical interactions.

**Methods:**

Analyzing with a CLECI codebook is a four-step process, i.e. preparation, codebook development, (double-)coding, and analysis and report. Core activities within these phases are research question formulation, data collection, selection of utterances, iterative deductive and inductive category refinement, reliability testing, coding, analysis, and reporting.

**Results and conclusion:**

We present step-by-step instructions for a CLECI analysis and illustrate this process in a case study. We highlight theoretical and practical issues as well as the iterative codebook development which combines theory-based and data-driven coding. Theory-based codes assess how relevant linguistic elements occur in natural interactions, whereas codes derived from the data accommodate linguistic elements to real-life interactions and contribute to theory-building. This combined approach increases research validity, enhances theory, and adjusts to fit naturally occurring data. CLECI will facilitate the study of communication form in clinical interactions and other institutional settings.

**Supplementary Information:**

The online version contains supplementary material available at 10.1186/s12874-022-01647-0.

## Introduction

The quality of communication between healthcare professionals (HCPs) and patients affects health outcomes. For example, positive (vs. negative) messages enhance patient recovery and decrease sensations of pain [1–3]. Many studies examine interactions with observational coding schemes like the Roter Interaction Analysis System (RIAS) [4] and the Verona Coding Definitions of Emotional Sequences (VR-CoDES) [5, 6]. These schemes consist of predefined categories that capture and quantify the content of communication between HCPs and patients to assess relevant communication phenomena such as the degree of patient-centered communication in homecare [7] or the association between a doctor’s response to patients’ emotions and visit duration [8]. Such observational coding schemes are effective in systematically summarizing relevant communication phenomena into cohesive and interpretable codes. The quantification of natural interactions helps to understand natural patterns of communication (e.g. when and how do patients voice their concerns) and to assess the relationship between specific communication phenomena and outcomes (e.g. the relationship between patient-centered communication and patient’s anxiety) [9].

Apart from communication *content* like positive messages, *form* is an imperative aspect of communication as well. The same message can be presented in different ways, e.g. benign test results can be presented as ‘the results look fine’ or ‘the results do not look bad’. While the message of both utterances is identical, their formulation differs. Such variations in form can elicit different outcomes in patients. For instance, compared to affirmative positive communication (‘the medicine is safe’), indirect positive communication (‘the medicine is not dangerous’) can increase patient anxiety and decrease adherence intentions and understanding of medicine use [10, 11]. Subtle differences in form also affect the course of doctor-patient interactions. General practitioners who ask whether there is ‘*some*thing else’ patients want to discuss evoke more follow-up responses from patients than when they ask whether there is ‘*any*thing else’ patients would like to discuss [12].

However, research on communication form is mainly experimental. Observational research of form is scarce and often qualitative in nature (e.g. [13, 14]). No well-defined coding protocols such as RIAS or VR-CoDES exist that systematically investigate variations in form, implying that patterns of form and how they may differ between groups remain unnoticed. Ultimately, little is known about how language use may systematically vary in everyday medical interactions and how this affects patient-reported outcomes. Therefore, we developed a coding protocol to quantitatively analyze variations in form.

CLECI (Coding Linguistic Elements in Clinical Interactions) – pronounced as ‘classy’ – enables the quantification of linguistic elements in medical interactions. Examples of linguistic elements are intensifiers or markers of uncertainty. CLECI is a theory- and data-driven observational method, which combines relevant theory-informed codes with potentially relevant linguistic elements that arise from observations of the interactions under analysis. Subsequently, linguistic elements are systematically analyzed to reveal communication patterns in real-life interactions [15], such as the use of intensified language by patients or markers of uncertainty by HCPs.

The aim of this paper is to describe the development of a codebook aimed at quantifying linguistic elements in clinical interactions. We present step-by-step instructions for the development, application, analysis, and reporting of the CLECI coding scheme, and we illustrate the methodological challenges related to the protocol using a case study [16].

## Methodology

The CLECI protocol has been developed for a research project analyzing linguistic markers by general practitioners (GPs) and patients in the context of medically unexplained symptoms, see [11, 16, 17] for the rationale and findings of these studies.

### Step-by-step-plan

**Fig. 1:**
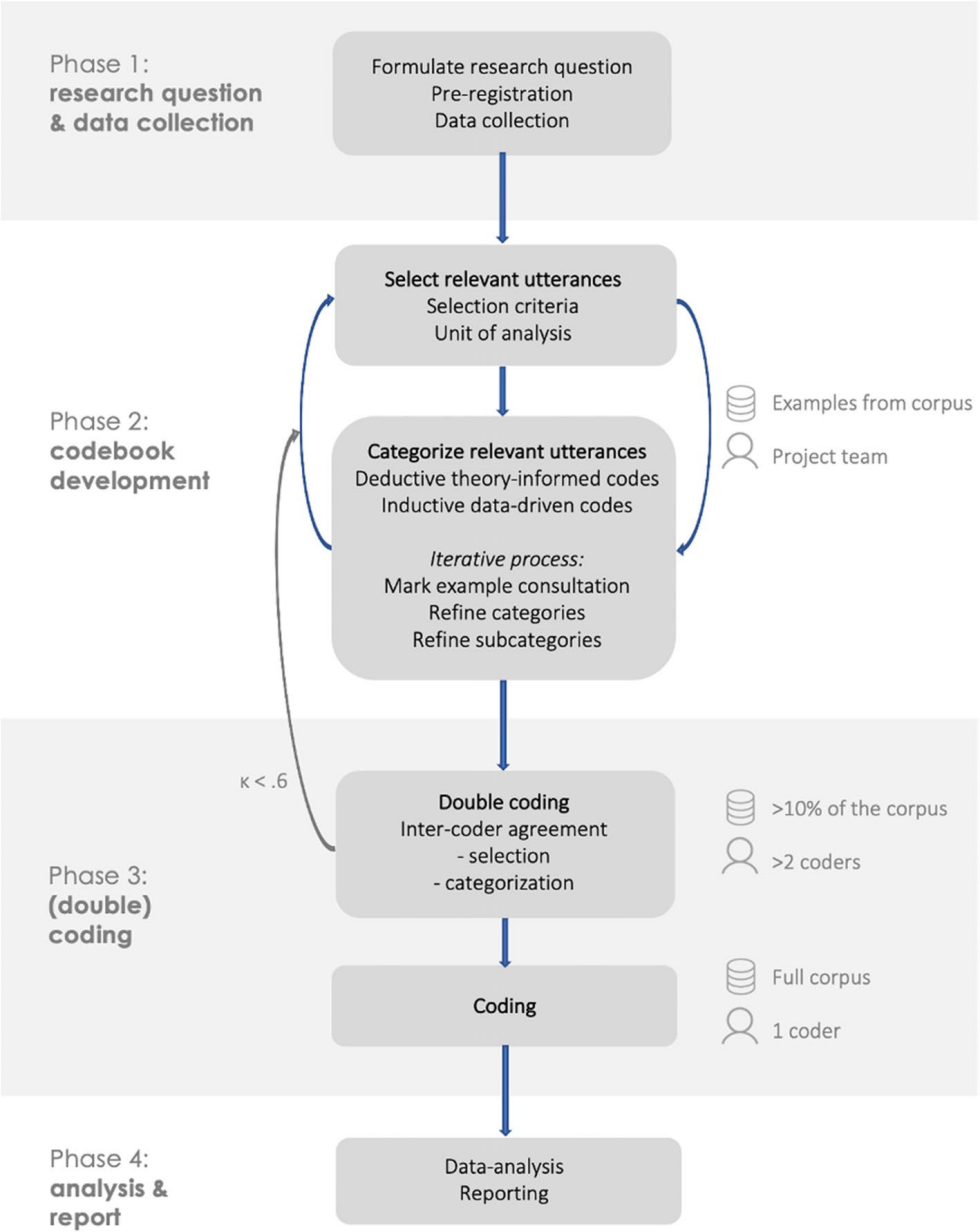
Visualization of the CLECI process

The coding process is divided into four phases, i.e. preparation, codebook development, (double-)coding, and, analysis and report. Figure 1 displays an overview of the phases and different accompanying steps. The preparation phase consists of multiple data-driven (inductive) and theory-informed (deductive) iterative cycles to develop a codebook that describes the selection and categorization of utterances. The third phase encompasses a double-coding procedure to calculate the reliability of the codebook, followed by the coding of the entire corpus. Lastly, the codes are analyzed and results are reported in the fourth phase.

### Phase 1 – research question and data collection

The first phase describes the preparatory steps required before codebook development, which include the joint formulation of the research question, data collection, and preregistration of the study (optional).

Research involving CLECI is aimed at the recognition and comparison of communication patterns of orally spoken data. Communication patterns are systematically recurring word formulations or language use. On their own, communication patterns offer little informative value as reference or control utterances are absent (e.g. patients using X number of negations in symptom descriptions). A comparative analysis, on the other hand, provides important insights into differences or similarities between various groups, e.g. patients with patients with non-epileptic seizures use more negations than patients with epileptic seizures. Differences in such linguistic elements can be used to predict a diagnosis [18]. CLECI, therefore, answers comparative research questions, i.e. questions that analyze differences between groups (between-subject design) or within one group over time (within-subject design or longitudinal research). Examples of research questions that can be answered with CLECI are presented in Table 1.

**Table 1:** Examples of research questions for CLECI

Research aim	Between groups – analysis of differences in communication patterns between two or more groups of people or between two or more types of consultations	Over time (longitudinal) – analysis of differences in communication patterns at different points in time
Examples of research questions	How do frequent GP visitors and occasional GP visitors differ in expressing anxiety about their health?	How have patients’ claims of epistemic authority changed in the last decade compared to 20 years ago (through the use of online health searching information)?
	To what extent does positive communication by the doctor differ in good versus bad news consultation?	How do patients’ pain and symptoms descriptions change during the course of a disease or illness?

Data collection follows the formulation of the research question and aim. CLECI can be used to analyze naturally occurring interactions, i.e. interactions “that would have happened regardless of the role of the researcher” [19]. Examples are doctor-patient consultations or (unedited) television interviews with medical experts. The rationale for using naturally occurring data is that patterns of language use are exposed as they occur in real-life [20]. Furthermore, naturally occurring data are not influenced by the researcher or the research aim. Researchers can analyze the data deductively while also inductively searching for unexpected or novel aspects that are not (yet) covered but do relate to the research aim [19].

Video-recordings give more insights into non-verbal behavior such as gaze or body posture compared to audio-recorded data. Since this type of information can help the interpretation and analysis of communication form, data are preferably recorded with video. For some research phenomena, however, audio-recordings also suffice (e.g. use of negations). The data are first transcribed verbatim following a Jefferson-lite style method by which additional interactional details such as pauses, pitch or interruptions are only transcribed if relevant to the research question (see [21] for an example).

It is recommended to preregister the study prior to data collection. Open science practices increase reproducibility and accessibility for academic and public audiences. This enhances discussion and implementation of research findings as well as collaboration among academics and participation of public audiences [22]. Specific theory-driven elements should be preregistered, while data-driven elements need further specification during the codebook development. Preregistration of the research questions and deductive concepts helps to specify the initial boundaries of the study. The clear distinction between predictions and postdictions prevents cherry-picking (see [23] for more information).

### Phase 2 – codebook development

Development of the codebook is divided into two stages, namely selection of relevant utterances followed by their categorization. In the first stage, coders define rules for exclusion and inclusion of utterances and the unit of analysis. In the second stage, rules on how to categorize utterances are formulated. All steps in phase 2 are subjected to an iterative process of deductive and inductive reasoning.

#### Selection of relevant utterances

Clinical interactions between physicians and patients cover a wide variety of topics beyond medical information. Selection criteria delineating relevant and irrelevant utterances ensure that the analysis corresponds to the research aim and question, e.g. selection criteria define HCPs’ utterances related to treatment when the role of language in treatment recommendations is researched.

Selection criteria are formulated in two interrelated steps. Firstly, coders mark all utterances related to the research aim using an exemplar consultation. Cases of doubt are collected and analyzed to (re)formulate coding rules and/or exceptions to the inclusion criteria, which are required to define the boundaries and limits of the research phenomenon. After discussions among coders, criteria are further specified and tested in another consultation. This process is repeated until doubts or differences between coders are case-specific and do not contribute to the formulation of generic coding rules.

Secondly, coders divide the utterances into units of analysis, allowing a systematic comparison between groups or over time. A unit of analysis is the smallest possible unit without losing its meaning [24]. As CLECI focuses on language use within specific contexts, grammatical finite clauses, i.e. clauses with one finite verb [10], will typically serve as the unit of analysis. Sentences containing multiple finite clauses, e.g. *I am tired because my headache kept me up*, are split up and analyzed separately. Contextual boundaries deviating from grammatical finite clauses as units of analysis can be defined if relevant for the research question. In this case, a turn-constructional unit, “the smallest interactionally relevant complete linguistic unit” [25], is commended as an alternative unit of analysis. It can consist of clauses without finite verbs (*too bad*), finite clauses (*I have a headache*), or whole sentences (*I think I have an ear infection*) [26]. Using turn constructional units as the unit of analysis allows a more flexible approach to the selection of relevant utterances. For instance, when studying uncertainty markers in patient utterances about symptoms, coders may need to include two finite grammatical clauses as one relevant utterance (e.g. “I think I have hay fever”). Similar to the formulation of selection criteria, units of analysis are applied and discussed until boundaries are mutually agreed upon by coders.

#### Categorization of relevant utterances

The second stage addresses the development of the coding categories. Coders construct or have constructed a preliminary codebook with categories and various sub-categories based on literature research in the preparation phase. The (sub-)categories cover any linguistic phenomena of interest, such as intensified language, language abstraction, or markers of uncertainty. The linguistic phenomena are translated into observable linguistic elements, see Table 2 for examples.

**Table 2:** Examples of linguistic elements for CLECI

Research phenomenon	Linguistic phenomenon	Linguistic element	Example
Exaggeration	Intensified language	Diminishers	A little, somewhat, a bit
		Intensifiers	Really, completely, particularly
Uncertainty	Uncertain language	Uncertain verbs	I think, it could
		Lexical items	Maybe, perhaps

Coders read exemplar consultations while focusing on three aspects:

1. deductive categorization. They examine whether the theory-based categories apply to the data, i.e. whether linguistic elements inspired by theory or taken from previous research occur in the data. Infrequent or absent categories are exempted from the codebook.
2. inductive categorization. Coders look for other possible (sub-)categories. If relevant to the linguistic phenomenon or research aim, they register linguistic elements not yet defined in the codebook, scan the literature for potentially relevant theories – if necessary – add these data-driven (sub-)categories to the codebook.
3. refinement of categories. Deductively and inductively developed categories are included in a revised codebook and assessed on four criteria: relevancy to the research aim, frequency in the data, whether they are mutually exclusive and exhaustive, and the extent to which they can be coded based on objective observations. Based on iterative assessments similar to the formulation of selection criteria and unit of analysis, coding (sub)categories are further refined or removed.

These three steps are repeated until no new categories or refinements arise from the data. Two aspects during category development require special attention, i.e. the number of categories and the extent to which examples are provided. These will be discussed below.

#### Number of (sub)categories

During the development of a codebook, coders make a trade-off between the quantity in main categories and subcategories. Coders decide upon the number of (sub-)categories depending on the research aim and theory. Research questions focusing on one or a few main categories require a detailed and elaborate analysis of a specific linguistic phenomenon (e.g. [27]). For instance, the analysis of HCPs’ expression of uncertainty during the diagnostic phase may be divided into subcategories such as explicit statements, modal verbs, lexical items, pragmatic particles, and conditional phrases. On the contrary, research questions covering multiple linguistic phenomena limit the extent to which they are subdivided into various subcategories. For instance, it is recommended to restrict the number of subcategories when analyzing various relevant linguistic markers in patients’ symptom descriptions (e.g. intensified, uncertain and abstract language versus uncertain language). A trade-off exists between the number of subcategories and reliability of coding; the more subcategories, the more complex the coding, which is likely to cause less agreement between coders.

#### Exhaustiveness of examples in categories

The codebook can describe categories in great depth with a list of examples taken from the data, or with general criteria that support coders to interpret and apply codes. Using a list of examples is objective and requires little to no interpretation from the coders, decreasing the likelihood of inconsistencies in the coding. A major drawback of this coding approach is that the example list must be exhaustive and complete. The lack of instructions accompanying the examples makes this approach inflexible, could create a tunnel vision for coders, and may result in potentially omitted relevant markers. A codebook using examples to illustrate rather than define coding categories allows a more flexible approach to coding. It can handle unique cases and irregularities that did not emerge during test coding sessions. A flexible codebook requires thorough training of coders and a deep understanding of the research aim, since coders are more likely to interpret the various (sub)categories in different ways.

If the categories are not clearly defined, over- or undercoding may occur. Overcoding occurs when coders incorrectly assign a category to a unit, e.g. ‘surprisingly’ is incorrectly coded as a diminisher in the utterance ‘the skin is surprisingly red’. Undercoding arises when coders overlook or miss instances of a certain category, e.g. a diminisher is omitted in the utterance ‘the skin looks red-*ish*’. Over- and undercoding can be minimized by providing concrete examples from the raw data and intensive training [28]. Intracoder reliability measures help gain insights into the extent of over- and undercoding [27]. These measures estimate the consistency of one coder in the coding process, thereby revealing which categories with low intracoder reliability may be unstable. To assess intracoder reliability, coders re-code a part of the initially coded dataset after 2 weeks. They calculate the reliability score similar to the intercoder agreement measures explained below. Coders discuss categories with low scores to explore discrepancies in the category description or interpretation of the coder and adjust the codebook accordingly.

### Phase 3 – (double-)coding

The third phase is divided into two steps, i.e. double-coding and coding. First, reliability of the codebook is assessed by calculating the agreement in the selection and categorization of relevant utterances among coders. When reliability is sufficient, the main coder proceeds to the next step of coding the entire corpus.

#### Double-coding

Consistent coding is imperative when qualitative data is quantified or (sub)groups are compared [29]. Consistency of coding among coders can be assessed with intercoder agreement (between coders, as opposed to within coders). The extent of agreement amongst coders is calculated separately for the identification and categorization of relevant utterances. As these steps are cumulative, coders reach a consensus about inclusion criteria before moving on to categorization.

Intercoder agreement is calculated by double coding a randomly selected subset covering at least 10 % of the entire corpus [30, 31]. For identification, a document is created containing all utterances from the subset, divided into separate units of analysis. Next, coders individually mark whether an utterance is relevant or not. Both relevant and irrelevant utterances are included to calculate intercoder agreement in the identification phase. If agreement is sufficient, the main coder selects all relevant utterances from the corpus to be categorized. For categorization, two or more coders individually code the selected subset of relevant utterances.

Intercoder agreement for identification and categorization are calculated with a reliability measure, e.g. Cohen’s Kappa, Scott’s Pi, or Krippendorff’s Alpha, see Popping [32] and Krippendorff [24] for an overview of the differences between reliability measures. For a more detailed description of how to perform an intercoder agreement analysis, see Burla et al. [29]. Interpretation of the measurement scores is presented in Table 3.

**Table 3:** Interpretation of reliability measure scores

Measurement score	Interpretation [33]	Action recommended
< .4	Insufficient	Examine differences between coders and refine boundaries of inclusion criteria and categories. Perform another round of double-coding on a new data subset.
.4 - .6	Moderate	Explore potential systematic differences between coders to further improve the codebook. Perform another round of double-coding on a new data subset. If the score remains > .4 and < .6, continue to coding. Present results with caution.
.6 - .8	Substantial	If desired, systematic differences can be explored.
> .8	Almost perfect	No

#### Coding

The development of the codebook is finished when coders attain a sufficient intercoder-agreement level. The main coder proceeds to the final step in which he or she codes the full dataset according to the final codebook. Coders are preferably blind to the condition, though a coder’s expertise does not always make it possible to do full blinding (e.g. coders with medical expertise may recognize the type of symptoms patients present). Since coding is based on transcripts rather than videos, coders are less prone to bias related to speaker characteristics such as age or gender.

Cognitive load (i.e. pressure on the coders’ capacity to process information) during the coding process should be limited to achieve reliable coding and to prevent over- and under-coding. Coders can choose to code categories horizontally (per utterance) or vertically (per category). Simultaneous coding is recommended when the coding of a specific category depends on another category. As an example, negations change the valence of an utterance (‘there is a need for a higher dose’ versus ‘there is no need for a higher dose’). Full transcripts are consulted when contextual information related to the utterance is required to decide upon the appropriate coding category. Finally, it is recommended to split the coding task into multiple sessions to prevent coding mistakes due to fatigue, and to mark cases of doubt and make a final decision at a later session.

### Phase 4 – analysis and report

The final phase describes the analysis of categorized utterances and reporting of the results.

#### Analysis

A final file for analysis is created after the main coder has coded all relevant utterances. We discuss two aspects regarding statistical testing, i.e. the model for analysis and hierarchical data (clustering).

The basic model for CLECI analysis is displayed in Table 4. In this model, linguistic elements (i.e. presence or absence per relevant utterance) serve as the outcome variable and comparison groups or different time points serve as predictor variables (e.g. comparing expressions of uncertainty markers before and after an intervention). Predictors and outcome variables may be reversed depending on the research question (e.g. [18]). The data for analysis is hierarchical, as the utterances occur within interactions, with specific HCPs possibly working at various institutions. Random intercepts should be tested and added to the research model whenever necessary, see [34, 35].

**Table 4:** Basic analytical model of CLECI assessing potential predictors of patterns of language use

Variable type	Variable content	Example
Outcome	Linguistic elements	Uncertainty markers, language abstraction, diminishers
Predictor	Comparison groups or points in time	Females & males, patients with medically explained & unexplained symptoms, before intervention & after intervention
Potential confounders	Pre-determined potentially relevant confounders	Age (patient and/or HCP), duration of interaction, years of experience of HCP

#### Reporting

The final step in the procedure consists of reporting the methods and results. A detailed description of the methodological process of the codebook development enhances reliability and encourages open science [28].

The results section should clearly distinguish between explorative and hypothesis-based analyses and discriminate between predictions and postdictions. In addition, researchers mention the stability of each category with regard to their respective Kappa’s as an indicator of how the results should be weighed. For instance, categories with Kappa’s above .8 can be regarded as stable, whereas Kappa’s below .6 should be interpreted with caution.

### Case study

Table 5 describes a case study illustrating the codebook development procedure of CLECI. This study aimed to compare linguistic elements in utterances of general practice patients presenting medically unexplained versus medically explained symptoms (see [16]). The aim of the case study is to illustrate the methodological considerations and challenges that accompany the CLECI protocol. The research question, data, and analysis (phase 1 and 4) are briefly described to provide background information, and we elaborate on particular challenges related to the codebook development and coding process (phase 2 and 3). We refer to the original publication for the theoretical background and findings of the study [16]. The complete codebook as used in the case study can be found in the Additional file 1.

**Table 5:** A case study illustrating the codebook development for CLECI [16]

**Phase 1: Research question and data collection**			
**Step**	**Example from Stortenbeker et al. (2022)**		
Research question	*“To what extent do linguistic markers in utterances differ between general practice patients presenting MUS and MES?”*		
Data collection	Verbatim transcripts of general practice consultations were derived from an existing research project [36].		
**Phase 2: Codebook development**			
**Step**	**Issue**	**Action**	**Example from Stortenbeker et al. (2022)**
Selection criteria	Inclusion and exclusion	Define research scope	Language use of patients presenting medically explained or unexplained symptoms to GPs.
		Read through training consultations	Patients talk about their past (‘but it was always low’) or current health problems (‘I am unstable’) as well as about potential future health issues (‘I think it could go wrong’).
		Redefine selection criteria	Scope was limited to include only utterances relating to current or past condition of patients, not prospective conditions.
Unit of analysis	Turn constructional unit	Define unit of analysis	Grammatical finite clauses served as unit of analysis in earlier stages.
		Read through training consultations	A more flexible unit of analysis was needed for subjectivity markers in cases such as ‘[I notice though] [that I’m getting sensitive to it]’.
		Redefine unit of analysis	Turn constructional unit was selected as the new unit of analysis.
Deductive categorization	Retain predefined category	Scan literature for relevant linguistic elements	Patients with MUS use more negations when describing (non-) occurrences of symptoms than patients with MES [37, 38].
		Formulate code	Negation – a) absent; b) syntactic; c) morphological
		Read through training consultations	Plenty of examples were found, such as ‘I am unstable’ and ‘I cannot move comfortably’, so negation was retained in the revised codebook.
Deductive categorization	Exclude predefined category	Scan literature for relevant linguistic elements	Doctors use more ‘illness terms’ (e.g. urination problems) towards MUS patients, whereas MES patients are often described with ‘disease terms’ (e.g. bladder infection) [39].
		Formulate code	Terminology – a) illness; b) disease
		Read through training consultations	Differentiating between the two was not easy (e.g. ‘I got dizzy’, ‘well then you’re all worn out’) and remained subjective. As an objective definition of the boundaries was not possible, the category was removed from the codebook.
Inductive categorization	Include category based on observations	Read through training consultations	Salient utterances such as ‘that ear keeps on whizzing’ were marked, suggesting ‘that ear’ operating as a separate agent as opposed to ‘I can hear pretty badly’.
		Scan literature for relevant studies	Patients can be disconnected from emotional and/or somatic experiences in various degrees [40].
		Formulate new code	Grammatical subject – a) first person (the patient, ‘I’); b) third person (patient’s biomedical or psychosocial state, ‘that ear’).
Iterative refinement	Add subcategory after test coding	Define code	Grammatical subject – a) first person; b) third person.
		Read through training consultations	Some utterances could not be indicated as having a first- or third-person subject, such as ‘[positive though] [that I do not have any new lesions]’ in which no subject is present in the first TCU.
		Redefine code	“empty subject” was included as a subcategory in the revised version of the codebook.
**Phase 3: (double) coding**			
**Step**	**Issue**	**Action**	**Example from Stortenbeker et al. (2022)**
Double-coding	Refine coding categories	Double code session	Intensity displayed a Kappa of .66.
		Explore systematic differences	One coder did not interpret certain time words as intensifiers, whereas the other coder did, e.g. ‘sometimes’, ‘all of a sudden’.
		Fine-tune codebook and coders	Remarks were added to the codebook. Words denoting an in- or decrease in time/frequency words are only marked when intensified such that ‘after that it was wrong again’ is not intensified, ‘all the time I think oh I’m getting tired’ is intensified.
Coding	N/A	N/A	Final coding was performed by the main researcher in various separate coding sessions. Cases of doubt were marked and evaluated at a later point in time.
**Phase 4: Analysis and reporting**			
**Steps**	**Example from Stortenbeker et al. (2022)**		
Analysis	Logistic binary random intercepts models with various linguistic markers as outcome variables, and consultation type (unexplained or explained symptoms) and codes related to message content as predictor variables, controlled for various relevant confounders.		
Reporting	Distinguished between hypothesis-based and explorative analyses. For more information, see Stortenbeker et al. (2022).		

## Discussion

This paper presented CLECI, Coding Linguistic Elements in Clinical Interactions, a protocol for the development of a quantitative codebook analyzing communication form in medical interactions. Communication form refers to *how* something is said in addition to *what* is said, such as communicating the safety of a medicine as ‘safe’ or ‘not dangerous’. Linguistic elements are categories of form, such as negations and intensifiers. It is important to study form in clinical interactions because variations in form can affect patients’ outcomes [10]. Yet, previous observation protocols focused on the content of communication, and studies assessing form have been mainly experimental rather than observational (e.g. [41, 42]). Since little is known about how linguistic elements are used in real-life clinical interactions, this paper introduced a carefully developed coding protocol to quantify communication form. CLECI codebooks follow a deductive and inductive development procedure. Theory-based codes serve to assess how relevant linguistic elements occur in natural interactions (deductive coding). On the other hand, codes derived from the data accommodate linguistic elements to real-life interactions and contribute to theory-building (inductive coding). This combined approach increases the validity of the research [28], enables theory-testing, and adjusts to naturally occurring data.

The systematic analysis of form in natural interactions facilitated by CLECI protocol has the power to reveal communication biases that are invisible to the naked eye. This is important since biases impact patient health outcomes (e.g. [43, 44]). CLECI is suitable for detecting implicit biases as these are communicated using specific linguistic elements, such as negations (see negation bias, [45]). Moreover, unlike experiments or interviews, CLECI is less likely to be affected by social desirability issues. When participants interact directly with researchers, they may display fewer biases in order to present a favorable image of themselves [46]. Socially desirable answering is less salient for participants as the data is unobtrusively gathered during natural interactions in which participants interact within their usual context and with authentic conversation partners instead of in a laboratory with a researcher [47]. Finally, CLECI can assess the degree to which biases are accurate. Patients with medically unexplained symptoms are, for example, expected to be vaguer in retellings of seizure accounts [18]. CLECI analysis of language can indicate whether this is indeed the case by systematically comparing patterns of abstract language between different groups [16].

Next to revealing communication patterns, CLECI can be applied for various other purposes. For example, CLECI can assess whether and how communication form affects patient outcomes. Quantitative observations of linguistic elements in natural interactions are, in this case, related to pre- and post-interaction measures such as patient anxiety or adherence intentions. To illustrate, HCPs who provide information about medical risks can induce anxiety in patients. Their level of anxiety may depend, however, on the statistical format used. When risks are described as “1 in 25”, patients perceive a higher likelihood of the risk to occur compared to when they are described as“4 in 100” [48]. Such variations in form may affect anxiety levels of patients. By combining a CLECI analysis of statistical risk formats (how risks are formulated) and measuring patients’ outcomes before and after the interactions (how anxious are patients about certain risks), experimental research is complemented with insights from real-life interactions, thereby endorsing external validity. Furthermore, CLECI can evaluate how communication training affects variations in form in medical interactions over time. For instance, analysis of positive language in interactions before and after positive communication training could assess whether HCPs communicate more positively after receiving the training. Finally, the CLECI protocol can be expanded to other institutional settings such as education or judiciary. Analysis of linguistic elements in educational interactions may provide insights in the effect of form on learning and memorization, whereas juridical interactions can be analyzed for potential biases in testimonial statements and court verdicts or the effect of form on understanding.

The CLECI protocol has some limitations. First, the local context of utterances is not taken into consideration. Data is unitized and aggregated to reveal overall patterns between various interactions. Since CLECI aims to analyze overall patterns of language use, no sequential coding takes place and form variations within a single interaction are not separately assessed. Consequently, utterances are not analyzed within their interactional context and may lose communicative meaning. For instance, when patients express uncertainty (‘I’m worried about my blood sugar levels’), HCPs can provide reassurance with intensified language (‘Your blood results in the past weeks have been particularly good’). In clinical interactions, the consultation phase – opening, history-taking, physical examination, diagnosis, plan, or closing phase – can be used as a proxy of how form changes during the progression of an interaction [16]. Second, between-group comparisons provide valuable insights into patterns of communication. Yet, groups are selected based on naturally occurring features rather than a controlled manipulation. Though statistical analyses allow to control for potential confounding, comparison groups may have features that cannot be detected or manipulated (e.g. when comparing communication form of patients with unexplained and explained symptoms, explained symptoms may have an unexplained component and vice versa). Third, the development of a codebook requires extensive time and resources, especially when inductive and iterative components are involved [49]. To reduce the time and effort needed for coding, automated natural language techniques can be used. These techniques tag words and utterances with, for example, their respective part-of-speech [50]. Automated coding can process large quantities of simple coding categories, which are in this case linguistic elements consisting of one word like negations or intensifying adjectives. Reliability of automated techniques is lower for more complex linguistic elements that require interpretation, such as coding utterance valence when negations are used (e.g. [51, 52]). Manual coding in addition to automated text processing is therefore necessary to guarantee consistent coding [53].

## Conclusion

Subtle differences in language can have a significant impact on patients’ outcomes. It is therefore important to analyze *how* (form) interactants communicate in addition to *what* (content) they are saying. Yet, existing coding schemes focus on the content rather than form of communication. This article has outlined the steps for developing a CLECI – Coding Linguistic Elements in Clinical Interactions – codebook and illustrates this process in a case study. CLECI is an observational and quantitative method for analyzing form in clinical interactions. The codebook development procedure combines theory-based and data-driven coding. This approach enables theory-building and theory-testing, and accommodates naturally occurring interactions, establishing research results with high external validity.

## Supplementary Information

**Additional file 1.**

## Data Availability

All data generated or analyzed during this study are included in this published article [and its supplementary information files].
