# Supplementary material for: Coding linguistic elements in clinical interactions: a step-by-step guide for analyzing communication form
Source: BMC Med Res Methodol. 2022 Jul 11;22:191. doi: 10.1186/s12874-022-01647-0 (PMC9277943; doi:10.1186/s12874-022-01647-0)
Supplement: Supplementary file 1 — Additional file 1. [file 12874_2022_1647_MOESM1_ESM.pdf]

CLECI

CODING LINGUISTIC ELEMENTS IN CLINICAL INTERACTIONS

Codebook for patients' language use

Inge Stortenbeker

Enny Das

Sandra van Dulmen

Tim olde Hartman

Wyke Stommel

Radboud University

Radboudumc
university medical center

Table of contents

Overview of codebook 3
Codebook 4
Overview of coding process 4
Double coding 4
PHASE 1 – Dividing the transcript into turns 4
Phase of the consultation 5
PHASE 2 – Identifying relevant utterances 6
Phase 2.1: Mark potentially relevant utterances 6
Quality of transcriptions and recordings 7
Unit of analysis 7
Phase 2.2: Select potentially relevant utterances 8
PHASE 3 – Categorising relevant utterances 8
1. Body/mind reference 8
2. Valence 9
3. Subject 10
4. Negation 10
5. Subjectivity 11
6. Intensity 11
7. Abstraction 12

Overview of codebook

PHASE 1: Dividing the transcript into turns

Divide the transcript into whose turn it is to speak. Number the turns and note the phases.

PHASE 2: Identifying relevant utterances

Inclusion criterion: The linguistic utterance is about the physical or psychological condition of the patient.

Unit of analysis: Turn constructional unit

- Phase 2.1: Mark relevant utterances
- Phase 2.2: Note down utterances in separate Excel file

PHASE 3: Categorising relevant utterances

1. Body/mind reference
   1) Biomedical
   2) Psychosocial
   3) Ambiguous

2. Content
   0) Neutral
   1) Positive
   2) Negative

3. Subject
   0) Empty subject
   1) Patient
   2) Situation

4. Negation
   0) Absent
   1) Syntactic
   2) Morphological

5. Subjectivity
   0) Absent
   1) CTMP
   2) Perception word
   3) Both

6. Intensity
   0) Absent
   1) Diminisher
   2) Intensifier

7. Abstraction
   0) Absent
   1) DAV/DV
   2) IAV/SAV
   3) SV
   4) ADJ

Codebook

Overview of coding process

The coding process consists of the following three phases:

- **Phase 1 – Dividing the transcript into turns:** Number the GP's turns and the patient's turns in chronological order. Read the transcript and note which phase of the consultation each turn takes place in.
- **Phase 2 – Identifying relevant utterances:**
    - *Phase 2.1:* Mark all relevant utterances made by the patient about their physical or psychosocial condition. Mark any doubtful cases in another colour.
    - *Phase 2.2:* Go through all relevant utterances and put them in a separate Excel file. Make a final decision about the doubtful cases.
- **Phase 3 – Categorising relevant utterances:** Code all utterances using the linguistic categories described in the codebook.

Double coding

For all phases, you calculate the inter-rater reliability by doublecoding 15% of the consultations (phases 1 and 2) and the corresponding utterances (phase 3). In phase 1, you compare the two coders' categorisation of the consultation phases. In phase 2 ,you write down all the structural units for the turns in a separate document and indicate whether each utterance is relevant and should be included. For phase 3, you calculate the inter-rater reliability based on the selection of the utterances included.

PHASE 1 – Dividing the transcript into turns

Convert a consultation's transcript into a table where each new turn is a new row in the table. Remove semicolons that are not written after the speaker's number. Select the entire transcript and go to Insert > Table > Convert text to table. Make sure your selection does not have blank lines. Select two columns and set semicolons as paragraph separators. After the table has been created, you add two extra columns to the right and a top row with the following titles (see Example 1). First, go through the transcript and remove verbal facilitator utterances such as "OK", "yes" or "hmm" that have been placed between a longer utterance from one of the two speakers. Only remove if 1) it was not steered by a previous turn (e.g. question or 'prompt') and 2) it does not interfere with the next turn in any way (see example 2). Merge cells if necessary. Then enter successive turn numbers for all turns by selecting the row and clicking 'Numbering'.

Example 1:

Speaker	Turn	Turn no.	Phase
1	So you've not been to the hairdresser?	1	1
2	No, not this time.	2	1
1	No, I don't think that's necessary. Only people who are clearly allowed, all people who look good? (10:02) Glad you're joining us, I'll have to show it to you in a bit. We're going to talk about your fainting spells and so on, and you'll be there too.	3	1
3	For the complete picture.	4	1
1	For the complete picture, yes.	5	1

You can see a fragment below where "oh?" is identified as a turn, while a "yes" later in the conversation is not treated as a turn. The "oh" in this example affects the subject of the next turn (namely "Yes, that"). The following "yes" also has a function in the conversation (it gives the speaker room to continue), but does not explicitly intervene with the turn. It should therefore be removed from the transcript and you merge turns 4 and 6 into one: 'Yes, that, I everything went well. And that's fine [...]".

Example 2:

Speaker	Turn	Turn no.	Phase
1	How's it going?	1	1
2	Well, for a while I had a, quite a session	2	1
1	Oh?	3	1
2	Yes, that, I everything went well.	4	1
1	Yes	5	1
2	And that's fine. People who keep stopping me like, you know, hey not so fast.	6	1

For each turn, note down what phase of the consultation the turn is in. It is possible for a transition between two phases to take place within one turn. In that case, write down both phases in the same column, separated by a dash.

Phase of the consultation

(*Source:* RIAS; Heritage & Maynard, 2006)

0) **Other:** The content of the turn has no relationship with any of the phases listed below.
1; "One moment please, it's 1 speaking" (GP answers the phone) or 2; "She gives a workshop and high tea for 25 euros"

1) **Opening:** The patient and GP greet one another and the doctor checks the reason for coming. The opening ends when a closed question by the GP marks the transition to the anamnesis.
   1; "Go ahead", 2; "Well, it's a long story."
2) **Anamnesis:** The GP uses various (closed) questions to find out the nature of the problem, personal and family circumstances, medical background and previous treatment, and other issues regarding the lifestyle or psychosocial circumstances.
   1; "Are you still experiencing dizziness?" 2; "Yes, still fatigue".
3) **Physical examination:** The GP carries out the physical examination. During the physical examination, the complaint or any other information may also be discussed, but the main activity in this phase is the examination itself.
   1; "This is just about the most painful place, isn't it?", 2; "Yeah, yeah".
4) **Diagnosis:** The GP discusses the diagnosis and explains it. During the diagnosis, a disease is identified or a working hypothesis is made. Utterances by the patient in reaction to this also count as the diagnostic phase.
   1; "The abdomen is looking OK, it just isn't doing what you want right now." 2; "No."
5) **Treatment recommendations:** The GP discusses the policy for the disease or complaints. This comes down to the action plan, the treatment, the therapy plan or follow-up appointments.
   2; "I can keep working and just do my thing?" – 1; "Yes, of course."
6) **Closing:** The consultation is concluded. The closing phase is often marked by utterances that mark a transition concluding the consultation.
   1; "We'll keep in contact, right?" – 2; "Yeah OK, thank you."

Comments

- Questions such as "How was your summer?" do not count as questions within the anamnesis as they do not mark the transition from 'Opening' to 'Anamnesis'.
- If the GP asks a closed question about themes as indicated under 'Anamnesis', the phase is referred to as 'Anamnesis'. This also applies if such a question is asked during other phases. If the question is about other subjects, the phase corresponding to the subject is used (e.g. the question "How long have you been taking sertraline?" is marked as the 'policy' phase if it is asked immediately after other utterances in that same phase).
- The 'physical examination' phase starts when the GP announces the examination and proceeds to carry it out, e.g. "I'm going to see what it looks like."

PHASE 2 – Identifying relevant utterances

Phase 2.1: Mark potentially relevant utterances

Mark all potentially relevant utterances by the patient. Mark any doubtful cases with another colour, and make a definitive decision about them in the next phase (Phase 2.2).

A language utterance is relevant if:

1. It is about the physical or mental condition of the patient. It pertains to their current situation or a situation that happened in the past.
2. The utterance contains an identifiable 'focal word' that makes clear that the linguistic utterance is about the patient's biomedical or psychosocial condition. (*Source:* Watson & Gallois, 2002)

Comments
- The patient's behaviour (whether health-related or not) is not included. For example, "I stopped drinking alcohol 14 days ago", "then I'm afraid to lie down".
- (Potential) future situations are not included, such as "I think it could go wrong".
- Utterances that are steered heavily by the GP are not included, for example: 1; "Does this hurt?" 2; "Yes, it hurts". This only applies to utterances that directly follow a question and are a clear repetition or variant on the GP's utterance. So 1; "Annoying or painful", 2; "It's sensitive" should be selected.
- Only select linguistic utterances if they are the patient's own assessment of their situation. Indirect speech such as "Then I call A, like, I don't feel well, getting sluggish" or "that's what P says too, I'm doing really well" are not included.
- Effects of physical examination or the treatment count as relevant utterances, for example, "I still have a mark from the blood test jab" or "and I think that [the medication] is working fine".
- If the utterance describes a cause or consequence of the complaint, it is only included if it refers to the patient's physical or emotional condition. "I did lose some weight" or "maybe that's because I don't feel so well" are included. If the explanation or the consequence does not directly describe the patient's condition, it is not included, e.g. "I'm on full sick leave", or "Where does it come from?"

Quality of transcriptions and recordings

If an utterance is potentially relevant, but the transcript is not good enough to correctly enter the coding, it is possible to listen to the recordings of the consultations again. For example: 1; "How's it going?" 2; "I had quite a session. Yes, that. I… Everything went well." ? (19:33) "and that's fine." This utterance takes place within the problem presentation and is potentially relevant for inclusion. However, the transcript has a few question marks where the transcriber was not sure of the utterance. In that case, you can listen to the original video again to try to refine the transcript. If there is still uncertainty about the transcription of the utterance after listening to the clip again, the utterance should not be included.

Unit of analysis

To guarantee the reliability of the coding, all selected sentences must be in a similar format, preferably as short as possible (Krippendorff, 2004). The unit of analysis in this study is a 'turn constructional unit' (TCU). A TCU is a coherent and independent utterance that is recognisable as "possibly complete" (cf. Clayman, Handbook of CA). It can consist of one or several words ("no pain"),

a clause ("last night I started stuttering") or a sentence ("well, this morning I measured a blood pressure of a hundred and seven"). It is important that an utterance only refers to one *item of interest*.

A sentence can consist of multiple TCUs that each have a separate description. For instance, "it's not so bad now but there's a certain wax there" forms one sentence but is split into two utterances. An utterance consisting of multiple TCUs is often marked with a comma or conjunctions such as 'and', 'or', and 'but'. This does not apply to utterances such as "I think that maybe it's a bit like migraine". Here, "I think" is not an independent utterance, and is included in that one TCU.

Comments

- Merged sentences where the finite verb form covers multiple objects, such as "Before that I did have some more problems with my stomach and my intestines", are split up (Watson & Gallois, 2002) into two separate units of analysis. The elements "my stomach" and "my intestines" describe two separate *items of interest* and are therefore treated separately.

Phase 2.2: Select potentially relevant utterances

Go through all relevant utterances and put them in a separate Excel file. Make a final decision about doubtful cases. Put each relevant utterance in a new row. For each utterance, note:

- The number of the utterance (chronological order);
- The number of the turn as indicated in Phase 1 of the coding process;
- The phase of the consultation as marked in Phase 1 of the coding process.

PHASE 3 – Categorising relevant utterances

For each utterance, analyse the items below. Code the utterances by indicating the correct category for each variable, using the corresponding number.

1. Body/mind reference
(Source: LCIAS; RIAS, Bekhuis, 2019)

1) **Biomedical:** The utterance is about the patient's physical condition, or how the patient thinks they are doing with regard to their physical condition.
   ''I have problems with my intestines".
2) **Psychosocial:** The utterance is about the patient's physical, emotional and/or social condition.
   "It's getting a bit too much", "I don't feel well", "It stresses me out".
3) **Ambiguous:** The utterance cannot be categorised unambiguously as biomedical or psychosocial. *"It starts like a sort of different perception", "I'm getting more sensitive to it", "There are nights I can't sleep".*

Comments

- The utterance must always have an identifiable 'focal word' that makes it clear that the utterance is about the patient's biomedical or psychosocial condition. It can be a verb ("but

then I almost faint"), noun ("I don't have a fever right now") or a deictic element referring to the condition ("It's too high now").

- If it is not immediately clear from the context whether the utterance is about the patient's biomedical or psychological condition, the context can be interpreted. For instance if the GP asks "How are you doing now?" and the patient answers "I'm doing better of course", within the context of the GP consultation and the remaining conversation (focusing on other physical complaints) it is most likely that this is an utterance about the patient's biomedical condition.
- Examples of psychosocial complaints are: descriptions of feelings, emotions, stress, general mood, philosophical viewpoints, values and convictions.
- The content of the utterance is described as 'ambiguous' if it is impossible to tell from the context if it is about a biomedical or psychosocial condition. For example, "But I really get it every day. Feeling light in the head and then wobbly on my feet. I'm unstable". Whereas the utterance "wobbly on my feet" is a biomedical description, it is unclear what "I'm unstable" refers to (unstable on their feet or an unstable psychosocial condition).

2. Valence

(*Source:* Stortenbeker *et al.*, 2018)

0) **Neutral:** The patient's physical or psychological condition is described from a neutral point of view, and it is impossible from the context to tell right away if there are positive or negative consequences to the physical or psychological condition.
   1; "That's good. And taking diclofenac again didn't have an effect on your bowel movements?"
   2; "I don't' think so, because I started taking it again and the result is the same." Or: "No ithe pulse rate is normal."
1) **Positive:** The patient's physical or psychological condition is positive or has positive consequences.
   "It's just wonderful."
2) **Negative:** The patient's physical or psychological condition is negative or has negative consequences.
   "That really bothered me".

Comments

- The categorisation of the valence can be directly based on the part of the utterance that indicates whether the utterance refers to a biomedical or psychosocial condition (e.g. "I'm also very tired"), or to an adjective or verb that characterises the condition (e.g. "that blood pressure is high").
- If the utterance does not clearly have a positive or negative valence, you analyse the context in which the utterance was made. For example, "Yes, I get that a lot" can refer to a positive or negative condition. In that case you look at the context to see what "that" refers to. If it is impossible to make this categorisation without interpreting what the speaker might have meant, an utterance is described as 'neutral'.
- For the category 'neutral', check properly if the utterance is directly about the patient's biomedical or psychosocial condition; only then is the utterance relevant.

- When assessing the valence, look at the meaning of the utterance. The assessment "It doesn't look bad" is indicated as positive content, expressed with a negation (category 5).

3. Subject

0) **Empty subject:** The sentence lacks a subject, or it is unclear what the subject refers to. This is the case when a sentence is split into two clauses where one does not contain a subject, or if the subject has a deictic element where it is unclear what it refers to.
   "On the plus side, I didn't get any new lesions" (split into two clauses: part 1 is marked as subject 'not applicable'); "nothing happened really".
1) **Patient:** The subject of the sentence refers to the patient themselves.
   "I have very poor hearing".
2) **State:** The subject of the sentence refers to the patient's biomedical or psychosocial condition.
   "That ear keeps ringing".

Comments

- If you have trouble determining the subject of the sentence, first look for the finite verb and make the sentence interrogative, e.g. "who or what keeps ringing?" > "that ear". If the answer to this question does *not* refer to the patient's condition (for example "it does help" > refers to a treatment and its effect), mark it as absent.
- In utterances that contain two clauses because of exceptions (e.g. they contain markers of subjectivity), select the subject of the sentence that the assessment of the biomedical or psychosocial condition is based on, e.g. "[I notice] that my left side doesn't feel right yet".
- If the utterance contains a self-correction, assume the grammatical subject that fits with the corrected utterance, e.g. "I… Everything went well".
- If the finite verb was left out of the utterance, then specify 'no subject', e.g. "of my joints" or "coughing a bit the day before".
- Impersonal 'you' as in "then you don't feel well" is noted as 'subject patient'.

4. Negation

(*Source:* e-ANS)

0) **Absent**
1) **Syntactic:** The utterance contains a negation that relates to the biomedical or psychosocial condition. In the explicit form, this is expressed by the negative adverbial qualifier 'not'. In the implicit variant, the negation is embedded in words that have a negative meaning such as none, nobody, never, nowhere (fusion with 'no') or less, without, etc. (fusion with other elements). No distinction has to be made between the implicit and explicit form.
   "That's not a good idea", "Doing that is never a bad idea".
2) **Morphological:** The utterance contains a prefix as a negation element. For example, *non*-smoker, *in*complete, *in*competent.
   "I feel very unstable inside".

3) **Both:** The utterance contains a syntactic and morphological negation, in other words it is a double negative.
"This doesn't make me unsteady".

5. Subjectivity
(*Source:* Bergqvist, 2018; van Beugen & van Schuppen)

0) **Absent**
1) **Complement-taking mental predicates:** The utterance contains words that indicate the speaker's point of view, such as I think, I realise, I believe, I assume, I understand, I notice. These are verbs that need a complement (continuation of a sentence) to become a meaningful utterance. (e.g. I think, I suppose, I believe, I reckon, I expect, I understand, I guess, I imagine, I realise).
"I've noticed that I am a bit sluggish".
2) **Perception words:** The utterance contains words that indicate the speaker's perceptions – sight, touch, smell and taste. It can refer to the direct, sensory observations, or be a referral to the speaker's internal state. E.g. *feel, smell, hear, see, taste*.
"I feel unwell".
3) **Both:** The utterance contains both a complement-taking mental predicate, and a perception word.
"I think, my shoulder, it feels like an inflammation".

Comments
- Words or expressions like "I hope that" (dynamic modality), or "I have to" (deontic modality) do not count as utterances of subjectivity.
- Only include explicit utterances of subjectivity in the sentence where the function is not ambiguous (or barely so). Examples are utterances such as "I notice that", "I feel that", "I think that". More implicit markers of subjectivity such as "you could say", where the meaning is multi-interpretable, are not included.

6. Intensity
(*Source:* Liebrecht, 2015; Liebrecht *et al.*, 2016; van Mulken & Schellens, 2006)

0) **Absent**
1) **Diminisher:** The meaning of the utterance is weakened by words such as 'small', 'a little', 'moderate' or diminutives. These barely change the meaning of the utterance.
"And then it starts bubbling a bit", "So that's going pretty well".
2) **Intensifier:** The meaning of the utterance is strengthened by one or more language elements. An intensifier indicates the extent to which the speaker's attitude is different from an unmarked situation, whereby the meaning barely changes.
"Everything, everything sounds very awful".

3) **Both:** The utterance contains both a diminisher and an intensifier.
   "I'm really almost vomiting".

Comments

- The qualification must be about the patient's condition, e.g. "My hands feel really bad". Utterances such as "the diastolic pressure, well, it can change every hour or so" do not count as a diminisher/intensifier.
- Language utterances are only marked as an intensifier if it is possible to create an alternative utterance where the intensifying utterance is left out (e.g. "it hurts *a lot"* > "it hurts") or replace it by a more neutral, weaker variant (e.g. "that looks *excellent"* > "that looks *good"*), without changing the positive or negative valence of the assessment as such. In the utterance "for three of four days, I couldn't go to the toilet sometimes", "sometimes" is not a diminisher because leaving it out would modify the meaning of the sentence (3-4 days not going to the toilet versus not going as often).
- Assess the meaning of the word in the context. "Not good at all" is indicated as an intensifier, but "not quite good" as a diminishing utterance. Sometimes, the intensification or diminution is indicated by multiple words, such as in "it went quite well the last few weeks". This is marked once as the presence of an intensifier (despite the fact that the words 'quite' and 'well' would have been marked separately as diminishers). The same applies to utterances such as "there's nothing I can do": "nothing" is a negation, but within this context it functions as an intensifier (the patient describes there's *nothing* she can do due to her complaints, but this is used stylistically rather than that she is fully incapable of doing anything).
- Intensifying adjectives such as 'excellent' may not have an additional adverb or adjective, but they do have an amplified meaning and can be replaced by 'good', for example. That is why they are also marked as 'intensifying'.
- Words that indicate an increase/decrease in frequency or time do *not* count as intensifying, e.g. "after that it happened again" (frequency), "and actually, for three or four days, I couldn't go to the toilet sometimes" (frequency), "so then all is well again for a while" (time). If the time element itself is intensified, it does count as an intensifier, e.g. "and you always feel that here in your throat", "I'm thinking the whole time oh I'm getting more tired now".

7. Abstraction

(*Source:* Coenen *et al.*, 2006; Schmid, Fiedler, Semin & Englich, undated)

0) **Absent:** There is no verb in the TCU.
1) **Descriptive action verbs (DAV) and descriptive adjectives (BA)**
   - A DAV refers to one specific action with a clear start and end and that can be unambiguously represented visually. It can also be seen as an objective description of an observable event.
     Fainting, sleeping, eating
   - DAs are adjectives that are associated with nomina (round, triangular, etc.), material adjectives (silver, plastic, etc.), colour adjectives (green, yellow, etc.) and physical nouns (people, animals, things, countries, cities).

"And I'm going grey", "Yes, but those heart palpitations... sometimes they don't go away", "I'd be panting like a horse" and *"I can really eat a lot again".*

- In metaphorical use of what at first seems to be a DAV, this is classed not as a DAV but as an IAV(2).
  "Well, well, those kinds of things I literally drop like a hot potato".

2) Interpretative action verbs (IAV) and state action verbs (SAV)
   - An IAV refers to an observable event in a situational context, but it requires additional interpretation. The verb does not refer to one specific visible action, object and/or situation, but has a context-dependent meaning. Because of this, it is impossible to make a simple visual representation.
   - An SAV is comparable to an IAV, except an SAV does not refer to an action in itself, but to an emotional consequence of an action (surprise, amazement, etc.).
     "Well none of that helps me", "That just isn't working".
3) State verbs (SV)
   - An SV refers to a permanent condition (feel, notice, hate). These verbs refer to subjective emotional or mental conditions that cannot be perceived by an observer. If the patient is the only person who knows precisely what is up, it is an SV.
   - Verbs of perception (see, hear) are often used; these actions cannot be controlled by the subject. When the perception is controlled (look, listen) it is an IAV. You can test this by asking if there is a deliberate action. If the answer is 'no', it is a state verb. This can also be tested by changing the sentence into the imperative. If it is not possible to change the sentence into the imperative, it is a state verb.
     "Yeah I feel nothing then", "so, this is the first time I've had it like this", "then, then, then, I do tick a lot of those boxes".
4) Adjectives (ADJ)
   - Adjectives/adverbs that indicate a property or attribute. It says how something is, not what something does or how it feels.
   - Nouns that refer to intangible things, such as: feelings, time-space, characteristics, events, imaginary people or things.
     "That's what's uncomfortable about it"; "Yes, at some point I get irritable".

Figure 1 depicts a schematic diagram of the coding process for adjectives and verbs as a measure of language abstraction.

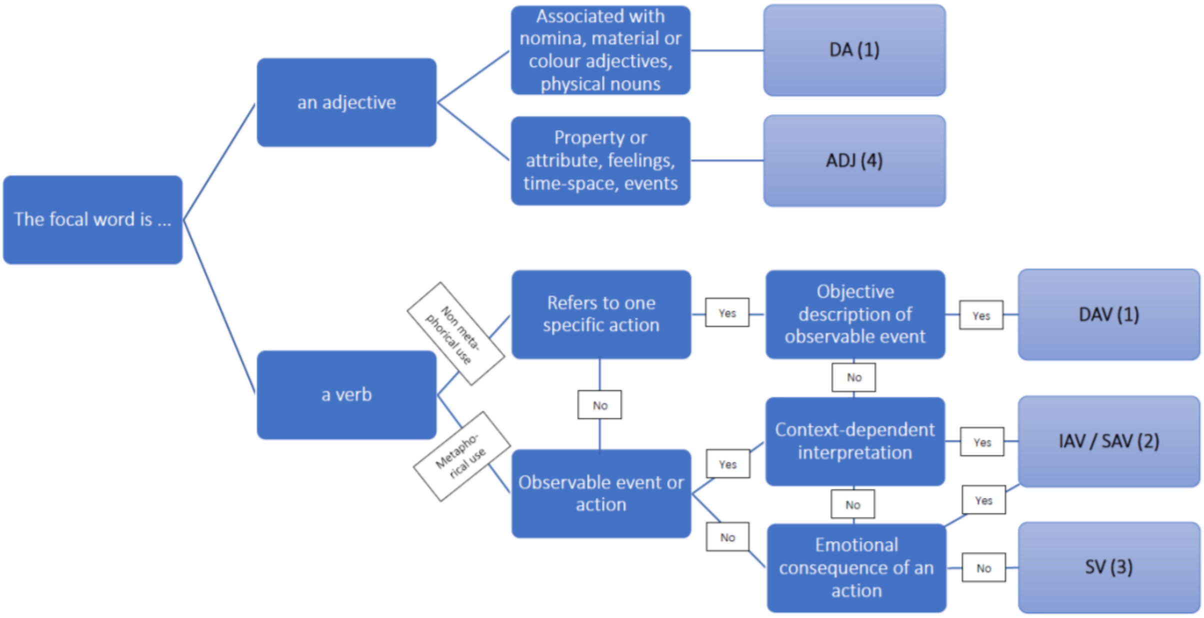

Figure 1: *Schematic diagram of the coding process in the linguistic category model*

<u>Comments</u>

- One *focal word* is selected. This is an independent verb or adjective. Linking verbs and/or auxiliary verbs may not be selected as the *focal word*. Only select an adjective if the sentence does not have an independent verb.
- The *focal word* can be determined by parsing the selected utterance using the following steps.
    - 1. <u>Select the independent clause</u> (if necessary): Dependent clauses give details about the patient's perspective or a detail of the condition. These are not included in the current coding.
    - 2. <u>Select a verb</u>: no verb = abstraction is absent.
    - 3. <u>Determine the verb type</u>: independent verb, auxiliary verb (be, have, become, will, can, may, must, want) or linking verb (be, become, seem, remain, appear, believe, occur – links the subject to a condition, function, capacity or property).
        - If the verb is not a linking or auxiliary verb: select as *focal word*.
        - If it is a linking or auxiliary verb: select adjective, noun or adverb as the *focal word*.

      Note: if there are multiple verbs, select the verb that best describes the condition.
